# Effects of BMPER, CXCL10, and HOXA9 on Neovascularization During Early-Growth Stage of Primary High-Grade Glioma and Their Corresponding MRI Biomarkers

**DOI:** 10.3389/fonc.2020.00711

**Published:** 2020-05-05

**Authors:** Wei Xue, Junfeng Zhang, Haipeng Tong, Tian Xie, Xiao Chen, Bo Zhou, Pengfei Wu, Peng Zhong, Xuesong Du, Yu Guo, Youyuan Yang, Heng Liu, Jingqin Fang, Shunan Wang, Hao Wu, Kai Xu, Weiguo Zhang

**Affiliations:** ^1^Department of Radiology, Daping Hospital, Army Medical University, Chongqing, China; ^2^Department of Neurosurgery, Daping Hospital, Army Medical University, Chongqing, China; ^3^Department of Pathology, Daping Hospital, Army Medical University, Chongqing, China; ^4^Department of Radiology, PLA Rocket Force Characteristic Medical Center, Beijing, China; ^5^Chongqing Clinical Research Centre of Imaging and Nuclear Medicine, Chongqing, China

**Keywords:** high-grade glioma, neovascularization, BMPER, CXCL10, HOXA9, PWI-MRI

## Abstract

Neovascularization is required in high-grade glioma (HGG). The objective of this study was to explore neovascularization-related genes and their corresponding MRI biomarkers during the early-growth stage of HGG. Tumor tissues from 30 HGG patients underwent perfusion MRI scanning prior to surgery were used to establish orthotopic xenograft models, pathologically analyze the tumor vasculature and perform transcriptome sequencing. The cases were divided into two groups based on whether the xenograft was successfully established. Microvascular density and BMPER, CXCL10, and HOXA9 expression of surgical specimens in the xenograft-forming group was significantly elevated and the microvascular diameter was significantly reduced, *in vitro* inhibition of BMPER, CXCL10, or HOXA9 in the glioma stem cell significantly suppressed its tube formation abilities. The *in vivo* experiment showed that BMPER was highly expressed in the early tumor growth phase (20 days), CXCL10 and HOXA9 expression was elevated with tumor progress, and spatially associated with tumor vasculature. Perfusion weighted MRI (PWI-MRI) derived parameters, rCBV, rCBF, K_trans_, and V_p_, were also increased in the xenograft-forming group. In conclusion BMPER, CXCL10, and HOXA9 promote early tumor growth and progression by stimulating neovascularization of primary HGG. The rCBV, rCBF, K_trans_, and V_p_ can be used as imaging biomarkers to predict the expression statuses of these genes.

## Introduction

Gliomas are the most common primary brain tumors in adults, accounting for over 70% of primary malignant brain tumors (1). Gliomas at World Health Organization (WHO) grades III and IV are referred to as high-grade gliomas and are highly malignant and progress rapidly. Although therapeutic strategies are constantly being developed, patients' prognoses remain poor (2).

Glioma neovascularization is required for tumor growth and closely related to tumor initiation, progression, metastasis, and relapse (3, 4). During the glioma early-growth phase, activated tumor cells are believed to first organize into cuffs around normal blood vessels to obtain nutrients (5) and generate massive tumor blood vessels by promoting endothelial cell proliferation by secreting vascular endothelial growth factor (VEGF), angiopoietin-2 (ANG-2), and stromal cell-derived factor 1 (SDF-1α) (6) and recruiting circulating endothelial progenitors (7) to provide essential nutrients for tumor growth and progression. Upregulation of the VEGF signaling pathway is the most important factor promoting neovascularization in gliomas. However, inhibiting VEGF only transiently suppresses tumor neovascularization and slows tumor growth but does not effectively improve glioma patients' overall survival (8, 9). This suggests that multiple angiogenic pathways play important roles in glioma neovascularization. For example, VEGF-independent endothelial transdifferentiation of tumor cells is another important approach by which gliomas acquire blood vessels (10). Therefore, other genes and pathways that promote glioma neovascularization must be investigated to progress treatment of gliomas. However, genes have specific temporal and spatial effects and usually play different roles during different tumor initiation and progression stages. The roles of neovascularization-related genes must be investigated during each stage of glioma progression to accurately treat patients and predict their prognoses.

Perfusion weighted magnetic resonance imaging is an effective means to non-invasively detect blood vessels and flow in tumors *in vivo*. Using the changes in signal intensity of the contrast agent in the blood vessels over time, dynamic susceptibility contrast (DSC)-MRI technology can calculate cerebral blood volume (CBV) and cerebral blood flow (CBF) (11). Dynamic contrast-enhanced (DCE)-MRI technology uses two compartment models and can calculate K_trans_, which reflects the permeability of newly generated blood vessels in the tissue, V_p_, which reflects plasma volume, V_e_, which reflects the volume of extracellular space out of the blood vessel, and K_ep_, which reflects the reflux rate of the contrast agent via the changes in contrast agent signal intensity over time in the blood vessels and extravascular spaces (12). These MRI parameters show changes in blood vessel structures and functions in the tumor region, which can be used to monitor the tumor response to treatment (13). These parameters are also related to the molecular characteristics and genetic phenotypes of tumors. A study confirmed that K_trans_ is related to the O6-methylguanine-DNA methyltransferase (MGMT) methylation status of glioblastomas (14) and relative CBV (rCBV) can predict the isocitrate dehydrogenase 1 (IDH1) mutation statuses in astrocytomas (15). Studies have also confirmed that mRNA expression levels are associated with MRI characteristics (16). These findings have important value for clinically and non-invasively screening specific gene expression within tumors.

Cell line tumor models have been widely applied in glioma research. However, as this research progresses, the consistency in stably passaging glioma cell lines and the authenticity reflecting the glioma's physiological and pathological features are questioned (17, 18). Compared with cell line tumor models, patient-derived orthotopic xenograft tumor models can more accurately reflect the histological and genetic properties of the original tumors and more realistically simulate the occurrence, progression and drug responses of the original tumor (19). Chi et al. screened third-generation mutation genes related to the malignant progression of IDH-mutant gliomas by comparing gene expression differences in the tumor tissues that could or could not generate a patient-derived xenograft (PDX) model (20). This finding provides new potential for targeted treatment of glioma patients. We found that in high-grade glioma surgical specimens, the microvascular density (MVD) was significantly increased, and microvascular diameters were significantly reduced in tumor tissues that could generate PDX models, suggesting that abundant neovascularization might play an important role in tumor occurrence and progression. Therefore, in this study, we screened neovascularization-relevant genes that were closely associated with early growth and progression in high-grade gliomas by analyzing the gene expression differences in tumor tissues that could or could not successfully establish PDX models. We also investigated whether imaging biomarkers could predict relevant gene expression. Our study provides new targets and novel detection ideas for antiangiogenic treatment against glioma.

## Materials and Methods

### Clinical Case Collection and Experimental Animals

After obtaining informed consent, this study collected 30 surgical specimens from 30 patients with primary high-grade glioma who underwent surgery at Daping Hospital during 2016.5–2017.10. Each tumor specimen was divided into three portions under sterile conditions. The first portion was used to extract primary tumor cells, which were used to establish orthotopic xenograft models. The second portion was embedded in paraffin to pathologically analyze the tumor vascular, and the third was used for transcriptome sequencing. Research was approved by the Human Research Ethics Committees of Daping Hospital, Army Medical University (Chongqing, China).

All non-obese diabetic-severe-combined immunodeficiency (NOD-SCID) nude mice used in this study were purchased from the Department of Experimental Animals (Daping Hospital, Army Medical University, Chongqing, China). All animal use protocols were performed according to the EU Directive 2010/63/EU for animal experiments and were approved by the Animal Use Subcommittee of Daping Hospital, Army Medical University.

### Magnetic Resonance Scanning and Data Processing of Human

MRI scans were performed on a 3.0 Tesla MRI scanner (Magnetom Verio, Siemens Medical Solutions, Erlangen, Germany) with an 16-channel head coil. The conventional MRI included axial and sagittal T1-weighted, T2-weighted, and axial fluid-attenuated inversion recovery (FLAIR) sequences. Sequence parameters were as follows: T1WI, TR/TE = 250/2.67 ms, SL = 5 mm, FOV = 230 × 230 mm; T2WI, TR/TE = 4,900/96 ms, SL = 5 mm, FOV = 230 × 230 mm; FLAIR, TR/TE = 8,000/94 ms, SL = 5 × 230 mm, FOV = 380 × 380 mm. DSC-MR imaging: The spin-echo echo-planar imaging parameters were as follows: TR = 1,500 ms, SL = 4 mm, TE = 30 ms, FOV = 230 × 230 mm, matrix = 128 × 128, FA = 90°, and NEX = 1.0. During 90 consecutive scans, 0.1 mmol/kg gadopentetate was injected through the basilic vein or median cubital vein at 4 mL/s at the 9th phase. DCE-MR imaging: Two sets of T1-weighted images were scanned with the T1-vibe sequence (TR/TE = 5.08/1.74 ms, FOV = 260 × 260 mm, matrix = 138 × 192, slice thickness = 5 mm, flip angle (FA) = 2° and 15°), and then 75 consecutive scans were performed using the T1-twist sequence (TR/TE = 4.82/1.88 ms, matrix = 138 × 192, slice thickness = 3.6 mm, FOV = 260 × 260 mm, FA = 12°) at a time interval of 5.3 s. At the 6th phase, the gadopentetate was injected via the basilic vein or median cubital vein at a rate of 4 ml/s and a dose of 0.1 mmol/kg. DSC-MRI was performed in 18 cases and DCE-MRI in 19 cases, seven of them underwent scanning of both sequences.

A Siemens syngo MR Workstation, and syngoMMWP software (version: VE36A) was used to analyze the DSC-MRI data. A compartment model was selected as the hemodynamic model (21), the arterial input function (AIF) was calculated using the middle cerebral artery, and the time and signal intensity curve of the brain were obtained by AIF. Then, the software calculated CBV and CBF map. The CBV and CBF values of tumor were quantifed as the average voxelwise of those parameters in whole tumor region, then CBV and CBF values of the contralateral healthy brain tissues were measured. The rCBV and rCBF values of the tumor were calculated from the ratio of the tumor area and healthy brain tissue.

DCE-MRI data were imported into a GE workstation, and OmniKinetics (version: 2.0) software was used for the analysis. The relaxivity of gadopentetate was 4.0 s^−1^mM^−1^ (r1 = 4.0) in our study, and it was used to control for the effect of tissue characteristics in the process of calculating K_trans_, V_P_, V_e_, and K_ep_ by the software. In order to calculate the T_10_ value of the voxel of the tumor and normal brain tissue (T_10_, the T_1_ value of the voxel before the injection, T_10_ = 1/R_10_). Multi flip angle (2° and 15°) scanning were performed before the multi-phase dynamic scanning. The specific calculation method was as follows: the signal value S_0_ was obtained from the T1 mapping scanning, the TR value of the sequence and the flip angleα was brought in equation S_(t)_ = S_0_(1–e^−[TR/T1(t)]^sinα)/(1–e^−[TR/T1(t)]^cosα), before injection, S_(t)_ = S_0_, so as to calculate T_10_, then R_10_ was calculated and the baseline value of R_10_ was obtained (22).

An Extend-Tofts model was selected as the hemodynamic model (23). The AIF was calculated by placing an ROI on the middle cerebral artery, and the time and signal intensity curve of brain were obtained from the AIF. According to the dynamic change of the signal intensity of tumor area before and after the injection of the gadopentetate, the software calculated K_trans_, V_P_, V_e_, and K_ep_ map. The K_trans_, V_P_, V_e_, and K_ep_ values of tumor were quantifed as the average voxelwise of those parameters in whole tumor region. All MRI data were measured by two radiologist who were blind to the experimental groupings.

### Orthotopic Xenograft Glioma Model Establishment and Glioma Stem-Cell Culturing

Fresh glioma tissue was rinsed with phosphate-buffered saline (PBS), cut into ~1-mm^3^ pieces, digested in papain (Worthington, USA) at 37°C for ~15 min, filtered through a 200-μm filter, and centrifuged at 300 g for 3 min. After discarding the supernatant, the pellet was resuspended in PBS so that the cell density was ~10^4^ cells/μl. Five microliters of cell suspension was aspirated with a micro-injector, and the needle was vertically inserted at 1.8 mm posterior and 2.2 mm to the right of the intersection between the midline and posterior canthus line of the brain in the NOD-SCID nude mice. The needle was first inserted to ~3.5 mm and withdrawn to 0.5 mm. The cell suspension was slowly injected (at ~1 μl/min), and the needle was withdrawn after ~10 min. The remaining cells were resuspended in Dulbecco's modified Eagle's medium (DMEM)/F-12 (Gibco, Carlsbad, CA, USA) supplemented with N-2 (Gibco), B-27 (Gibco), epidermal growth factor (EGF; 20 ng/ml; Sigma, USA) and basic fibroblast growth factor (bFGF; 20 ng/ml; Peprotech, USA) and cultured in an incubator containing 5% CO_2_ at 37°C. The orthotopic xenograft models from each patient was established in 5 mice (male, 4–6 weeks), a total of 150 mice were used. Mice were randomly assigned by the staffs of department of experimental animals who were blinded to the experimental design and the animal numbers were chosen by a statistical power calculation. All animals entered into the procedures survived to the end, gave analyzable data, and were actually included in the analyses.

### The Monitoring of PDXs in NOD-SCID Mouse

Tumor growth was monitored using a Bruker 7.0T small animal MRI imager, BioSpec 70/20 USR (Bruker, Ettlingen, Germany). The scanning sequence was TurboRARE-T2 WI (TR/TE = 4,000 ms/45 ms, FOV = 25 mm × 25 mm, thickness 0.5 mm). Magnetic resonance scanning was performed every 2 weeks for the first 3 months after the injection of primary tumor cells, and then about once a month. Mice were performed a magnetic resonance scanning and sacrificed when they showed clinical signs that determine the impending death, such as emaciation, weakness or spinal curvature. If no xenograft was observed at the endpoint, it was concluded that PDX was not established.

### Transcriptome Sequencing and Screening of Differentially Expressed Genes

Glioma tissue was freshly collected for transcriptome sequencing (Wuhan Seqhealth). The surgical specimens were divided into two groups, a xenograft-forming group and a non-xenograft-forming group, according to whether they formed xenograft in the NOD-SCID nude mouse brains. Differences in mRNA expression between both groups were compared. In detail, RNA was extracted from the glioma tissue using TRIzol reagent (Invitrogen, USA). After removing the rRNA and double-stranded RNA, the RNA was reverse-transcribed into double-stranded cDNA. Polymerase chain reaction (PCR) was performed to amplify and establish the RNA library, which was inspected for quality. After passing the quality inspection, the RNA library was sequenced on an Illumina sequencer. To analyze the differentially expressed genes, gene expression levels were determined by reads per kilobase per million reads (RPKM) and subjected to sample biological repeat correlation testing. Differentially expressed angiogenesis-related genes were screened between the xenograft-forming and non-xenograft-forming groups (fold change >2, *P* < 0.05).

### Immunohistochemical Staining and Blood Vessel Quantification

Immunohistochemical staining were performed as described previously (24). The antibodies used was raised in rabbits against human CD34 (Abeam, Cambridge, UK). Two-micron-thick serial sections were used for immunohistochemical staining after dewaxing in xylene. Antigen retrieval was performed in a boiling EDTA solution (pH 9.0) for 2.5 min, and then slices were washed with PBS after natural cooling. H_2_O_2_ (10%) and goat serum were used to block endogenous peroxidase activity and non-specific antigens, respectively. Each slice was incubated overnight with solution containing the primary antibody at 4°C. Specimens were then washed with PBS and incubated with HRP-conjugated goat anti-rabbit secondary antibodies at 37°C for 30 min. DAB was used to visualize antigens.

Five fields were randomly selected under 100× light microscopy. The number, diameters and areas of the CD34-positive lumens were measured, and the average values were used as the tumor microvascular density, diameter, and area for each case. All pathological data for the tissues were measured by two highly experienced staff members who were blind to the experimental groupings.

Paraffin-embedded xenograft were subjected to hematoxylin and eosin (HE) and immunohistochemical staining. The antibodies used was raised in rabbits against human CD34, GFAP, BMPER (bone morphogenetic protein endothelial cell precursor–derived regulator), CXCL10 (chemokine ligand-10), and HOXA9 (homeobox A9) (Abeam, Cambridge, UK).

### Immunofluorescence Staining of Glioma Stem Cell Spheres

We successfully cultured two cases with glioma stem cell spheres from the glioma surgical specimens: DP3321 and DP7857. The glioma stem cell spheres were cultured in suspension and plated onto poly-L-lysine (PLL)-coated 96-well plates. After removing the medium, the stem cell spheres were fixed in 4% paraformaldehyde for 20 min, permeabilized in 0.3% Triton X-100 for 10 min, blocked in 5% bovine serum albumin for 1 h, and incubated in corresponding primary antibody at 4°C overnight. After washing with PBS, the samples were incubated in the dark at room temperature for 2 h and then incubated in 4′, 6-diamidino-2-phenylindole (DAPI) for 5 min. After washing with PBS, the samples were observed under fluorescence microscopy. CD133, Nestin, Sox2, and GFAP antibodies (Abcam, Cambridge, UK) were used for immunofluorescence staining.

### *In vitro* siRNA Inhibition Experiment

Glioma stem cell spheres were digested with 0.05% trypsin to single-cell suspensions, and 3 × 10^5^ cells were plated into each well in 6-well plates containing 2 ml of culture medium. Cells were cultured in an incubator containing 5% CO_2_ at 37°C for 12 h. Intracellular BMPER, CXCL10, and HOXA9 expression was suppressed with siRNA (Ribobio, Guangdong, China), respectively. The siRNA target sequence for BMPER was GGAGAGATGTGGTCCTCTA, for CXCL10 was GTCCACGTGTTGAGATCAT and for HOXA9 was TCCTCCAGTTGATAGAGAA. Cells were divided into five groups: siBMPER, siCXCL10, siHOXA9, negative control (NC), and blank control (BC). Five microliters of 20 μM siRNA (Ribobio) stock solution was diluted with 120 μl of 1× riboFECT™ CP Buffer (Ribobio) and mixed gently. Next, 12 μl of riboFECT™ CP Reagent (Ribobio) was added to the diluted siRNA solution, mixed gently by pipetting, incubated for 0–15 min at room temperature and added to the already prepared 6-well plates. After mixing, cells were cultured in the incubator. After 48 h, part of the cells were used for real-time quantitative PCR (qRT-PCR) and western blots to determine the effects of siRNA inhibition. The remaining cells were used to determine their tube formation capacity.

### RNA Isolation and qRT-PCR

Total RNA was extracted using TRIzol reagent (Invitrogen, USA). A Nanodrop 2000 spectrometer was used to determine RNA concentration. One microgram total RNA was used to synthesize cDNA with a Transcriptor First Strand cDNA Synthesis Kit (Roche, Switzerland) after the removal of residual DNA with a TURBO DNA-free Kit (Thermo, USA). Subsequently, qRT-PCR was performed using FastStart Essential DNA Green Master Mix (Roche, Switzerland) and the LightCycler 96 System (Roche, Switzerland). The expression level of genes was analyzed and normalized to GAPDH. The fold change in gene expression was evaluated using the 2^−ΔΔ*CT*^ method. The sequences of the PCR primer pairs are as follows: BMPER, forward 5′- AAGAGTGCCTCCTACGAGTG-3′ and reverse 5′-CTGCCTTTCACACAAGCACA-3′; CXCL10, forward 5′-GTGGCATTCAAGGAGTACCTC-3′ and reverse 5′- TGATGGCCTTCGATTCTGGATT-3′; HOXA9, forward 5′-ACTTTGTCCCTGACTGACTATG-3′ and reverse 5′- AGGGTCTGGTGTTTTGTATAGG-3′; GAPDH, forward 5′-CTCCTCCACCTTTGACGC-3′ and reverse 5′- CCACCACCCTGTTGCTGT-3′.

### Western Blot Analysis

Protein extraction and western blots were performed as described previously (25). Cells were washed with cold PBS and lysed with RIPA buffer. Samples were quantifed using a protein assay (Micro BCA; Termo Fisher Scientific) and subjected to SDS/PAGE. Then the same amount of protein was transferred to polyvinylidene difluoride membranes and probed overnight at 4°C with antibodies for BMPER, CXCL10, HOXA9, and GAPDH (Abcam), respectively. After incubation with horseradish peroxidase-conjugated secondary antibodies, blots were visualized with an enhanced chemiluminescence detection kit (Roche Diagnostics, Basel, Switzerland).

### Tube Formation Assay

Matrigel (Corning, Bedford, USA) was thawed, and tips and 96-well plates were precooled at 4°C. The Matrigel was diluted with PBS at a 1:1 ratio and added to precooled 96-well plates at 55 μl per well. The plates were incubated in an incubator containing 5% CO_2_ at 37°C for 1 h. Glioma cell spheres transfected with siRNA were digested with 0.05% trypsin and resuspended in DMEM supplemented with 10% fetal bovine serum. Cells were added to Matrigel-coated 96-well plates at 5 × 10^4^ cells per well and cultured in an incubator containing 5% CO_2_ at 37°C for 6 h. Five fields were randomly selected under 100× light microscopy, and blood vessel-like structures were counted. The average number was used as the number of blood vessels in the cells. The blood vessel-like structures were counted by two experienced staff members who were blinded to the experimental groupings.

### Monitoring the Blood Vessel-Related Gene Expression in PDXs

The *in situ* tumor model was established using DP3321. Tumor growth was monitored using a Bruker 7.0T small animal MRI imager, Biospin 70/20 (Bruker, Ettlingen, Germany). The scanning sequence was TurboRARE-T2 WI sagittal view (TR/TE = 4,000 ms/45 ms, FOV = 25 mm × 25 mm, thickness 0.5 mm). Tumor growth curves were calculated from the largest cross-section of the tumor in the sagittal view. Xenograft specimens were collected at the early stages (20, 30, and 40 days) and late stage (80 days) of tumor growth and were stained for GFAP, CD34, BMPER, CXCL10, and HOXA9 to determine the relationships between BMPER, CXCL10, and HOXA9 expression at different tumor stages and tumor neovascularization.

### Statistical Analysis

SPSS 19.0 (IBM, Armonk, NY, USA) was used for the statistical analysis, and data are represented as the means ± SD. The data with normal distribution (Shapiro-Wilk test) and homogeneity of variance (*F*-test) were tested by two sample *t*-test, otherwise by wilcoxon rank sum test. The diagnostic threshold (cutoff value) was acquired from a receiver operating characteristic (ROC) curve to assess differential neovascularization-related gene expression in the tumors. Image features and pathological results reproducibility were assessed using the intraclass correlation coefficient (ICC). A value of *P* < 0.05 was considered significant and *P*-values were adjusted for multiple comparisons by Bonferroni-adjusted.

## Results

### Establishment of Patient-Derived Orthotopic Xenograft Glioma Models

**Figure 1:**
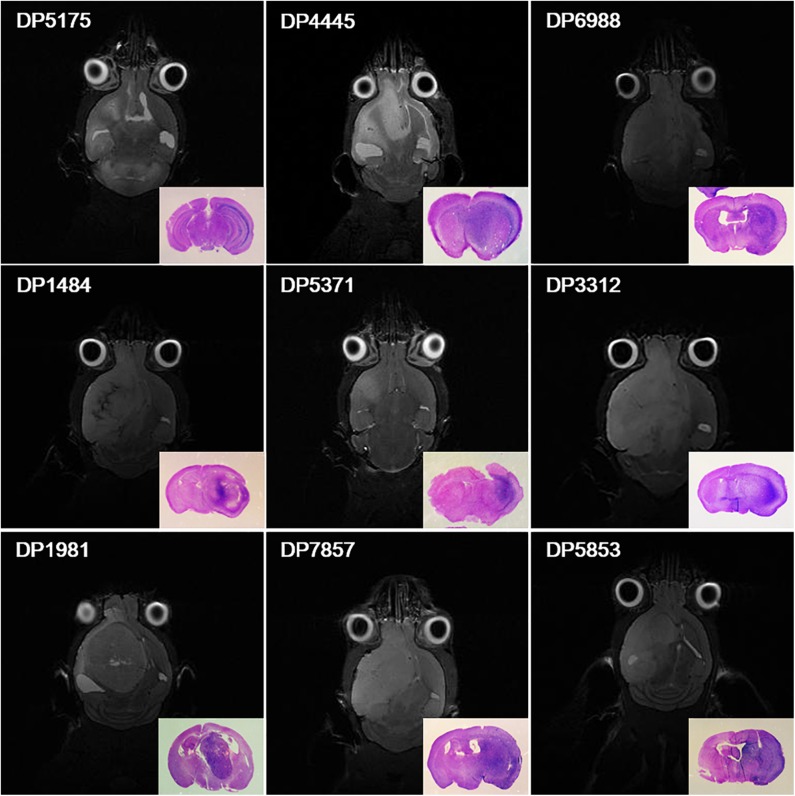
Orthotopic xenograft glioma models were established for 9 cases. Magnetic resonance cerebral coronal T2-weighted imaging and HE staining of the corresponding axial sections of the brain tissues (bottom right corner) showed that primary glioma cells from 9 cases formed xenografts in NOD-SCID nude mice.

Surgical specimens from 30 patients with primary high-grade gliomas were collected (14 cases of WHO grade III gliomas and 16 cases of WHO grade IV glioblastomas). Orthotopic xenograft glioma models were successfully established from 9 cases. Totally, the PDX was established in 45 mice (9 × 5 = 45) and the PDX was not established in 105 mice (21 × 5 = 105) (1 case of WHO grade III glioma and 8 cases of WHO grade IV glioblastoma) (Figure 1).

### Relationship Between the Microvascular Status of the Glioma Surgical Specimens and the Ability to Form Orthotopic Xenograft Glioma Models

**Figure 2:**
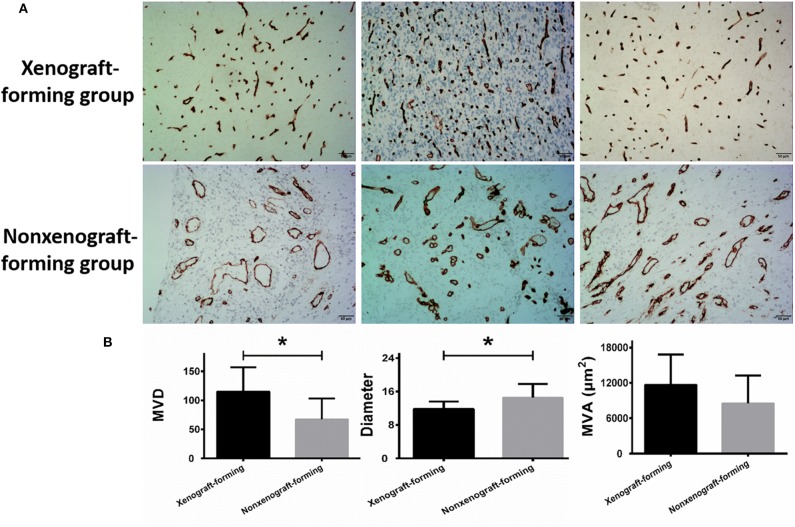
Microvascular analysis of glioma surgical specimens. **(A)** CD34 immunohistochemical staining, the upper row represents CD34 staining of 3 surgical specimens in the xenograft-forming group, and the lower row represents CD34 staining of 3 surgical specimens in non-xenograft-forming group. **(B)** Microvascular analysis of 9 surgical specimens in xenograft-forming group and 21 surgical specimens in non-xenograft-forming group. Compared with the non-xenograft-forming group, the microvessels from the tumor surgical specimens of the xenograft-forming group had higher microvessel density (MVD) and smaller diameters. The microvascular area (MVA) did not differ significantly between the two groups. Data are presented as the mean±SD. *P < 0.05.

The ability of glioma surgical specimens to form xenografts was associated with microvascular density and diameter in the tumor tissue. Tumor tissues that could form xenografts had a higher microvascular density (*P* = 0.003) and a smaller mean microvascular diameter (*P* = 0.019), however, no association was found between the ability of glioma surgical specimens to form xenografts and microvascular area (95% CI: −735.960–7533.477) (Figure 2; Table 1). The reproducibility of the histopathological results were assessed using the intraclass correlation coefficient (ICC = 0.924).

**Table 1:** Histopathological analysis of the glioma surgical specimens.

	**Xenograft forming group (*n* = 9)**	**Non-xenograft forming group (*n* = 20)**	***P*-value**
MVD	114.96 ± 41.97	67.49 ± 35.68	0.003
Diameter	11.86 ± 1.73 μm	14.60 ± 3.24 μm	0.019
MVA	11634.08 ± 5172.79 μm^2^	170157.30 ± 4715.86 μm^2^	0.082

*Data are represented as the means ± SD, MVD, microvessel density; MVA, microvessel area; n, number*.

### Differential Expression Analysis of Neovascularization-Related Genes in Surgical Specimens Between the Xenograft-Forming and Non-xenograft-Forming Groups

In the two glioma surgical specimen groups, the expression levels of BMPER, CXCL10 and HOXA9 in the surgical specimens that could form xenografts were significantly higher than those in the surgical specimens that could not form xenografts. However, no significant differences were detected in the expression of genes that promote tumor angiogenesis (VEGF, PDGF, SDF1, and HIF1) (Table 2). We used immunohistochemical staining to evaluate the activation of VEGF. VEGF was positively expressed in tumor vascular hotspots in both groups, but there was no significant difference in the area of the VEGF positive region between the two groups (Supplementary Material 1).

**Table 2:** Differential expression analysis of neovascularization-related genes in surgical specimens between the xenograft-forming and non-xenograft-forming groups.

**Geneid**	**logFC**	**logCPM**	**F**	***P*-value**	**FDR**
BMPER	1.104299	4.126899	4.594521	0.042844	0.99998
CXCL10	2.152519	2.675148	11.5653	0.002446	0.99998
HOXA9	3.923925	−0.3877	12.30047	0.001888	0.99998
VEGFA	0.266265	8.863766	0.153259	0.699033	0.99998
VEGFB	0.034804	5.481137	0.012242	0.925046	0.99998
VEGFC	−0.87295	1.500243	2.302993	0.14844	0.99998
VEGFD	1.220408	−0.91077	3.987051	0.067468	0.99998
ADGRB1	0.015033	6.569727	0.000373	0.984766	0.99998
ADGRB3	−0.00398	6.423576	5.56E-05	0.994113	0.99998
BAIAP2	−0.04885	5.297582	0.020926	0.896956	0.99998
HIF1α	0.120036	8.581223	0.114733	0.739837	0.99998
DLL4	−0.18645	4.159931	0.133605	0.718052	0.99998
NOTCH1	−0.3361	8.453163	0.855924	0.364433	0.99998
NOTCH2	0.012841	8.667439	0.001159	0.973132	0.99998
NOTCH2NL	0.143784	6.511975	0.15956	0.693236	0.99998
NOTCH3	−0.48754	7.738713	0.78887	0.383608	0.99998
NOTCH4	−0.06755	3.128933	0.027537	0.889718	0.99998
ANG	−0.25013	1.835498	0.142394	0.709362	0.99998
PDGFA	−0.4534	5.692098	0.546723	0.467117	0.99998
PDGFB	−0.06942	4.917269	0.022345	0.882471	0.99998
PDGFC	−0.21335	5.56359	0.419721	0.564307	0.99998
PDGFD	−0.70391	3.78351	0.850256	0.366022	0.99998
PDGFRA	−1.752	10.29704	3.209511	0.086339	0.99998
PDGFRB	−0.41728	7.351284	0.528063	0.474731	0.99998
SDF1	−0.06775	4.623607	0.009082	0.9249	0.99998
FGF1	−1.19343	7.463494	3.048296	0.094126	0.99998
MMP2	0.407572	6.600393	1.842536	0.233055	0.99998
MMP9	−1.52058	5.705015	2.061249	0.164506	0.99998

*P < 0.05 Representative genes were up-regulated in the xenograft-forming group (fold change >2)*.

### *In vitro* Inhibition of BMPER, CXCL10, or HOXA9 Expression Reduced the Angiogenic Capacity of Tumor Cells

**Figure 3:**
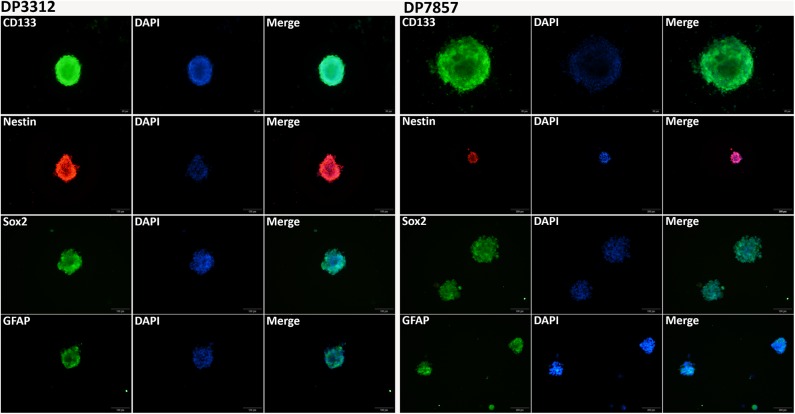
Immunofluorescence staining of the glioma stem cell spheres. Immunofluorescence staining of DP3321 **(left)** and DP7857 **(right)**, from two glioblastoma cases, showed that the stem cell markers CD133, Nestin, and Sox2 and the astrocyte marker GFAP were all positive.

**Figure 4:**
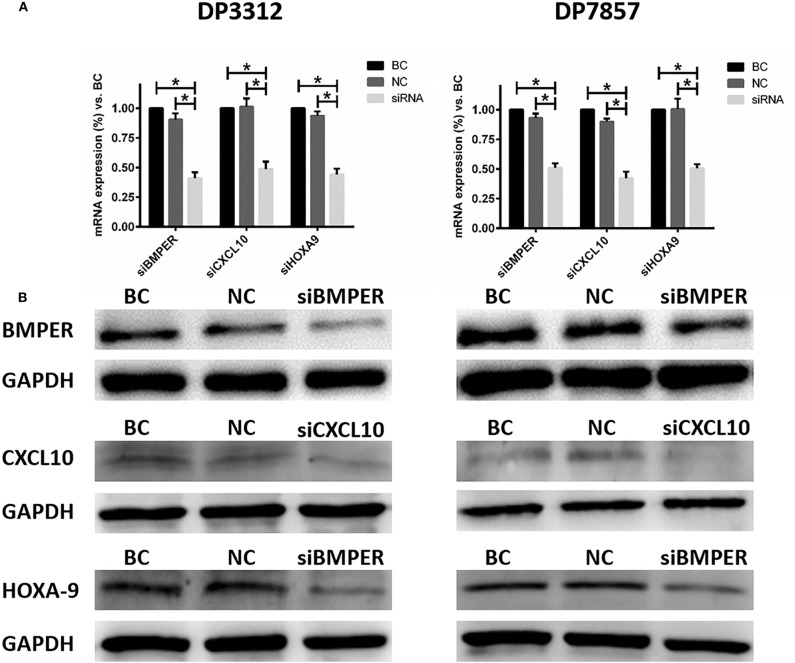
Suppressing BMPER, CXCL10, and HOXA9 expression in the glioma stem cell spheres from cases DP3312 (left) and DP7857 (right) by siRNA. **(A)** Examination (qRT-PCR) of the mRNA expression of corresponding genes after suppression by siRNA. BC, blank control; NC, negative control. Data are presented as the mean ± SD. *P < 0.05. **(B)** Examination (western blot) of corresponding protein expression after suppression by siRNA.

**Figure 5:**
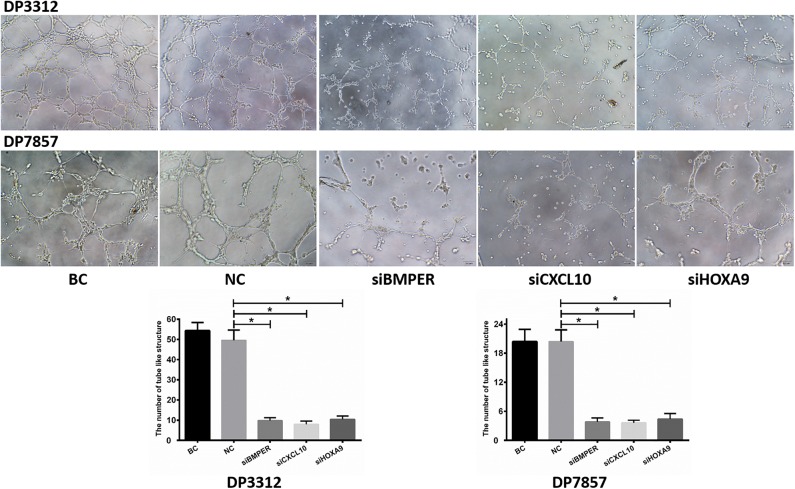
*In vitro* tube formation assay. Suppressing BMPER, CXCL10, and HOXA9 expression in the glioma stem cell spheres from cases DP3312 and DP7875 by siRNA significantly reduced the tumor cells' *in vitro* tube formation ability. BC, blank control; NC, negative control. Data are presented as the mean ± SD. *P < 0.05.

Immunofluorescence staining of the 2 cases of primary tumor stem cell spheres (DP3321 and DP7857) showed that the glioma tumor cell marker GFAP and the glioma tumor stem cell markers CD133, Nestin, and Sox2 were all positive (Figure 3), confirming that the extracted primary cells were stem cells originating from gliomas. We inhibited the expression of BMPER, CXCL10, and HOXA9 using siRNA in the 2 cases of glioma stem cell spheres. The inhibition efficiencies were detected by WB and qRT-PCR (Figure 4). Compared with the black control group, the siBMPER, siCXCL10, and siHOXA9 expression levels in DP3321 were 46.32, 49.71, and 43.64%, respectively, and the siBMPER, siCXCL10, and siHOXA9 expression levels in DP7857 were 53.21, 47.46, and 46.31%, respectively. After inhibition, the tube formation capacities of DP3321 and DP7857 were significantly reduced (Figure 5; Table 3). Consistency in the measurement results was determined using the intraclass correlation coefficient (ICC) test, with ICC = 0.963.

**Table 3:** *In vitro* inhibition of BMPER, CXCL10, or HOXA9 expression reduced the ability of tumor cells to form tube like structure.

	**DP3312** **(*n* = 5)**	**Negative control** **(*n* = 5)**	***P*-value**	**DP7857** **(*n* = 5)**	**Negative control** **(*n* = 5)**	***P*-value**
siBMPER	9.8 ± 1.48	49.6 ± 5.03	0.000	3.8 ± 0.84	20.4 ± 2.41	0.000
siCXCL10	8 ± 1.58	49.6 ± 5.03	0.000	3.6 ± 0.55	20.4 ± 2.41	0.008
siHOXA9	10.4 ± 1.67	49.6 ± 5.03	0.000	4.4 ± 1.14	20.4 ± 2.41	0.000

*Data are represented as the means ± SD, and represents the number of tube like structure. N, number*.

### Expression of BMPER, CXCL10, and HOXA9 in the Orthotopic Xenograft Glioma Models

**Figure 6:**
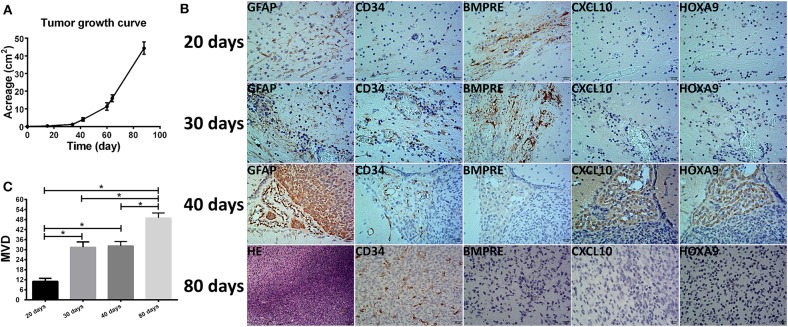
Relationship between the tumor microvessels in the orthotopic xenograft glioma models and BMPER, CXCL10, and HOXA9 expression. **(A)** Tumor growth curve of primary glioma stem cell sphere DP3312 after *in situ* xenografting in NOD-SCID nude mice. **(B)** Tumor microvessels during different xenograft growth stages and their relationships with BMPER, CXCL10, and HOXA9 expression. On day 20 of tumor growth, BMPER was highly expressed, while CXCL10, HOXA9 and tumor vascular marker CD34 were poorly expressed. On day 30, BMPER was highly expressed, CXCL10 and HOXA9 were poorly expressed, and CD34 expression was elevated. On day 40, BMPER expression was reduced, and CCXL10 and HOXA9 expression was elevated, indicating a spatial association with CD34 expression. In the tumor end stage, BMPER, CXCL10, and HOXA9 were all poorly expressed, while CD34 was highly expressed. **(C)** Microvascular densities of the xenograft at different growth stages (days 20, 30, 40, and 80). The value of *P* < 0.008 was considered significant after Bonferroni-adjusted. *P < 0.05.

We used DP3321 to establish orthotopic xenograft models in NOD-SCID nude mice. The first day after model establishment was used as the first day of tumor growth to calculate the tumor growth curve (Figure 6A). The relationships between BMPER, CXCL10, and HOXA9 expression and the tumor blood vessels were determined in the early- (20, 30, and 40 days) and late-tumor growth stages (80 days) (Figures 6B,C). On day 20 of tumor growth, BMPER was highly expressed, while CXCL10, HOXA9 and the tumor blood vessel marker CD34 were lowly expressed. On day 30, BMPER was highly expressed, CXCL10 and HOXA9 were lowly expressed, and CD34 was elevated. On day 40, BMPER expression was reduced, while CXCL10 and HOXA9 expression was elevated, which was spatially correlated with CD34 expression. In the late-tumor growth stage, BMPER, CXCL10, and HOXA9 were all lowly expressed, while CD34 was highly expressed.

### Relationship Between Perfusion MRI Derived Parameters of Patients and the Ability to Form Orthotopic Xenograft Glioma Models

**Figure 7:**
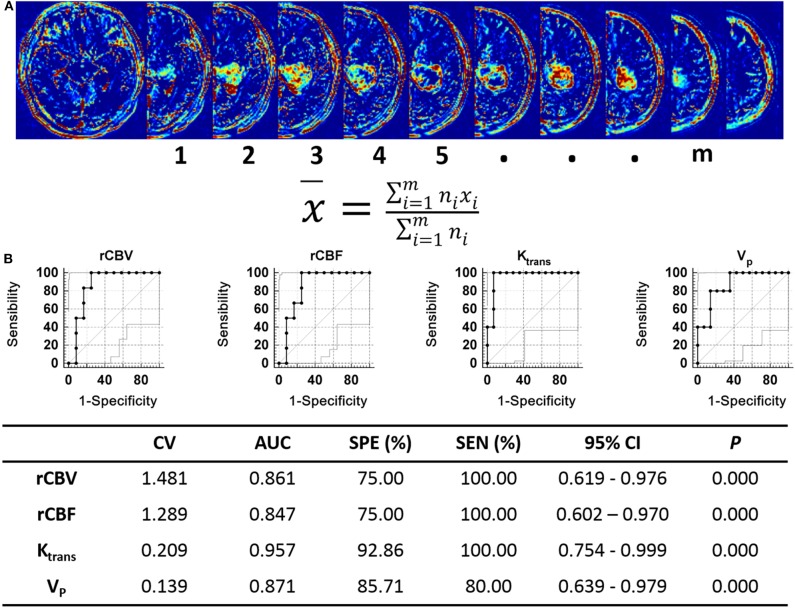
Analysis of perfusion MRI-derived parameters in human. **(A)** The K_trans_, V_P_, V_e_, K_ep_, rCBV, and rCBF values of tumor were quantifed as the average voxelwise of those parameters in whole tumor region. The tumor areas of each slices were selected as the ROIs, x represents the values of K_trans_, V_P_, V_e_, K_ep_ rCBV, or rCBF of each ROI, n represents the number of voxel included in ROIs, m represents the number of ROIs. **(B)** ROC curve analysis of the perfusion MRI scanning parameters with significant differences between the xenograft-forming group and the non-xenograft-forming group.

We analyzed the perfusion MRI derived parameters in the 30 cases and measured the DSC-MRI derived parameters rCBV, rCBF, and the DCE-MRI derived parameters K_trans_, V_p_, V_e_, and K_ep_ in each tumor case. Consistency in the measurement results was determined using the intraclass correlation coefficient (ICC) test, with ICC = 0.874. Our results showed that the DSC-MRI parameter rCBV, rCBF, and the DCE-MRI parameters K_trans_ and V_p_ of cases that could form xenografts were significantly higher than those of cases that could not form xenografts (*P* = 0.014, *P* = 0.018, *P* = 0.001, *P* = 0.003, respectively) (Table 4). A ROC curve was used to further analyze the diagnostic efficacy of tumor rCBV, rCBF, K_trans_, and V_p_ in determining whether the tumor tissues could form xenografts. The optimal diagnostic threshold of rCBV in determining whether the tumor tissues could form xenografts was 1.481, the area under the curve was 0.861, and the sensitivity and specificity were 100.00 and 75.00%, respectively. The optimal diagnostic threshold of rCBF in determining whether the tumor tissues could form xenografts was 1.289, the area under the curve was 0.847, and the sensitivity and specificity were 100.00 and 75.00%, respectively. The optimal diagnostic threshold of K_trans_ in determining whether the tumor tissues could form xenografts was 0.209, the area under the curve was 0.957, and the sensitivity and specificity were 100.00 and 92.86%, respectively. The optimal diagnostic threshold of V_p_ in determining whether the tumor tissues could form xenografts was 0.139, the area under the curve was 0.871, and the sensitivity and specificity were 80.00 and 85.71%, respectively (Figure 7). Transcriptome sequencing results confirmed that the expression levels of BMPER, CXCL10, and HOXA9 in the cases that could form orthotopic xenograft glioma models were significantly higher than those in the cases that could not form xenografts (Table 2). Therefore, the perfusion MRI scanning parameters rCBV, K_trans_, and V_p_ can be used as imaging biomarkers to predict BMPER, CXCL10, and HOXA9 expression and further guide treatment.

**Table 4:** The differences of perfusion MRI derived parameters between xenograft forming group and non-xenograft forming group in human.

	**Xenograft forming group**	***N***	**Non-xenograft forming group**	***N***	***P*-value**
rCBV	2.25 ± 0.47	6	1.43 ± 0.82	12	0.007
rCBF	1.77 ± 0.29	6	1.19 ± 0.69	12	0.014
K_trans_	0.50 ± 0.33 min^−1^	5	0.17 ± 0.07 min^−1^	14	0.001
k_ep_	0.49 ± 0.32 min^−1^	5	0.41 ± 0.47 min^−1^	14	0.219
V_p_	0.25 ± 0.11	5	0.10 ± 0.07	14	0.002
V_e_	0.63 ± 0.05	5	0.47 ± 0.18	14	0.130

Data are represented as the means ± SD. N, number.

## Discussion

Abundant neovascularization is an important feature in high-grade gliomas. The newly generated tumor blood vessels have incomplete structure and function (26), leading to the specialized tumor microenvironment characterized by hypoxia, low PH and high interstitial pressure, which is closely related to the glioma's immunologic escape and treatment resistance (27). However, although anti-angiogenic treatments targeting multiple proangiogenic factors have achieved some effects, the preset goals remain unmet. This is mainly because glioma neovascularization is a complicated pathological process regulated by multiple factors and pathways whose expression levels are temporally and spatially specific (4). Early glioma diagnosis and treatment can significantly improve patients' prognoses (28). Therefore, investigating neovascularization-related molecules during the early growth phase of glioma has important significance for antiangiogenic glioma treatment.

In this study, we collected surgical specimens from 30 cases of high-grade gliomas and extracted primary tumor cells, which were then injected into the brains of nude mice to simulate the early growing process of activated tumor cells in the brain. Our results showed that orthotopic xenograft glioma models were successfully established from surgical specimens from 9 cases. Pathological analysis of the blood vessels from the surgical specimens of these 30 cases showed that the specimens that formed xenografts had more active neovascularization, their microvascular density was significantly increased, and their microvascular diameters were significantly reduced. These results indicate that neovascularization plays an important role in the early growing process of high-grade gliomas.

Previous studies have confirmed that glioma tumor stem cells are similar to normal neural stem cells and have the potential of self-renewal and multidirectional differentiation (29). They can transdifferentiate into endothelial cells and release multiple cytokines and chemokines to promote angiogenesis (30, 31). In glioblastomas, up to 60% of endothelial cells have the same genetic mutations as tumor cells, suggesting that many tumor blood vessels originate from tumor cells (32). Some studies reported that tumor cell transdifferentiation to endothelial cells is induced by VEGF and VEGF receptor 2 (VEGFR2) (33). However, some studies have also reported VEGF-independent transdifferentiation (10), which is one reason why anti-VEGF signaling-pathway drugs cannot extend glioma patient survival. In return, endothelial cells and tumor vasculature also play an important role in the self-renewal and tumorigenesis of cancer stem cells (34). Through transcriptome sequencing analysis of glioma surgical specimens, this study found that genes that promote tumor angiogenesis, such as VEGF, PDGF, FGF, and HIF1α, were not differentially expressed between the two tumor specimen groups, but the BMPER, CXCL10, and HOXA9 expression levels in the surgical specimens that could form orthotopic xenografts were significantly higher than those in the specimens that could not form orthotopic xenografts. In the *in vitro* experiments, we found that primary glioma stem cell spheres (DP3321 and DP7857) had extremely high tube formation capacities and that inhibiting BMPER, CXCL10, or HOXA9 expression significantly reduced the angiogenic capacities of the tumor cells, indicating that these three genes can promote tumor-derived neovascularization in primary high-grade gliomas obviously.

Previous studies showed that BMPER, CXCL10, and HOXA9 promote tumor angiogenesis and tumorigenesis in lung cancer, colon cancer, basal cell carcinoma, ovarian cancer, and liver tumor (35–38). Other studies also showed that CXCL10 and HOXA9 expression levels were elevated in some glioma tissues (39, 40). In our study, tumor growth curves of xenografts were analyzed to confirm that in the early stages of tumorigenesis, BMPER was highly expressed, while CXCL10, HOXA9, and CD34 were lowly expressed. Later, BMPER was continuously highly expressed, which significantly increased CD34-positive blood vessels in tumor regions. Along with further tumor progression, BMPER expression was gradually reduced, while CXCL10 and HOXA9 expression was gradually elevated, which was spatially associated with CD34 expression. These results indicate that in the early-growth stage of primary high-grade glioma, BMPER promotes tumor growth by promoting tumor angiogenesis, and, along with further tumor progression, CXCL10 and HOXA9 expression levels are gradually increased, thus playing a role in promoting tumor growth by promoting tumor angiogenesis. Therefore, BMPER, CXCL10, and HOXA9 can be used as novel targets for antiangiogenic treatment of primary high-grade gliomas.

Accurately detecting target gene expression levels is the key to individualized and targeted tumor treatments. However, gene sequencing is invasive, time-consuming and costly; thus, popularizing it in clinical practice is difficult. Therefore, accurate, reliable, convenient and non-invasive biomarkers must be determined to reflect target gene expression. Previous studies have confirmed that both DSC-MRI and DCE-MRI can non-invasively reflect the blood flow and structural and functional vascular changes in the tumor region (41), and correlate with the molecular characteristics and genetic phenotypes of tumors (42). In this study, we found that the DSC-MRI parameter rCBV, rCBF, and the DCE-MRI parameters K_trans_ and V_p_ in cases that could form orthotopic xenograft glioma models were significantly higher than those in cases that could not form orthotopic xenograft glioma models. Increases in both rCBV, rCBF, and V_p_ indicated increased blood flow volume and increased K_trans_ indicated increased vascular permeability in the tumor regions in cases that formed xenografts. The newly generated tumor blood vessels have high permeability due to their incomplete structure. Therefore, increased K_trans_ also indirectly reflected abundant neovascularization in the tumor region. The differences in the above perfusion MRI derived parameters between the xenograft-forming and non-xenograft-forming groups were consistent with the vascular tissue pathological analysis, confirming the accuracy of the MRI scanning. Some of the values of V_e_ and V_p_ were physiologically implausible in our study, it was more likely that the assumption of Extended-Tofts model as a well-mixed compartment was not valid, especially in tumor tissue, suggesting that the Extended-Tofts model may not be completely valid in this setting and more applicable pharmacokinetic models should be studied. ROC curve analysis indicated that the scanning parameters rCBV, rCBF, K_trans_, and V_p_ of the DSC-MRI and DCE-MRI can be used as imaging markers to predict the expression statuses of BMPER, CXCL10, and HOXA9 in tumor tissue, which can guide the antiangiogenic treatment of primary high-grade gliomas.

We followed 30 patients with primary high-grade glioma. Four were lost to follow-up, and 1 died of other causes. Among the remaining 25 patients, the ability of the tumor tissue to form an orthotopic xenograft glioma model was not significantly associated with the patients' prognoses. This outcome may be due to the different postoperative therapeutic strategies among patients. Among the patients we followed, few underwent routine radiotherapy or chemotherapy after surgery. Most underwent no standard treatments, and a few received no treatment after surgery. Therefore, clinical studies with larger sample sizes are required to determine the association between patients' prognoses and the BMPER, CXCL10, and HOXA9 expression statuses, which was a limitation of this study. Further study about the mechanisms by which these three genes regulate neovascularization, and their relationship with VEGF or other neovascularization related pathways will be the next step of our research. Additionally, changes in tumor growth and progression after inhibiting BMPER, CXCL10, and HOXA9 expression in the xenografts should also be studied.

In summary, in primary high-grade gliomas, BMPER, CXCL10, and HOXA9 expression can promote early-phase tumor growth and further progression by increasing tumor neovascularization. Of these, BMPER mainly played a role in the early-growth phase of tumors, while CXCL10 and HOXA9 mainly played roles in tumor progression. These findings provide novel targets for antiangiogenic glioma treatment. We also found that the DSC-MRI and DCR-MRI scanning parameters rCBV, rCBF, V_p_, and K_trans_ could be used as imaging biomarkers to non-invasively predict BMPER, CXCL10, and HOXA9 expression in tumor tissue, which provides an effective means of diagnosing, treating, and monitoring primary high-grade glioma.

## Data Availability Statement

The original contributions presented in the study are publicly available. This data can be found here: the NCBI Gene Expression Omnibus (GSE148292).

## Ethics Statement

The studies involving human participants were reviewed and approved by Ethics Committees of Daping Hospital, Army Medical University. The patients/participants provided their written informed consent to participate in this study. The animal study was reviewed and approved by Ethics Committees of Daping Hospital, Army Medical University.

## Author Contributions

WZ and XC conceived and designed the experiments. WX and JZ performed the experiments and wrote the first draft of the manuscript. BZ, YY, and HL performed clinical case collection. XD and YG performed the MRI scanning. JF and SW performed MRI data post-processing and analysis. PW and KX performed surgical samples collection. PZ and HW performed histopathological analysis. HT and TX performed transcriptome sequencing.

## Conflict of Interest

The authors declare that the research was conducted in the absence of any commercial or financial relationships that could be construed as a potential conflict of interest.
