# Supplementary material for: Effects of BMPER, CXCL10, and HOXA9 on Neovascularization During Early-Growth Stage of Primary High-Grade Glioma and Their Corresponding MRI Biomarkers
Source: Front Oncol. 2020 May 5;10:711. doi: 10.3389/fonc.2020.00711 (PMC7214627; doi:10.3389/fonc.2020.00711)
Supplement: Supplementary file 1 [file Table_1.DOCX]

**
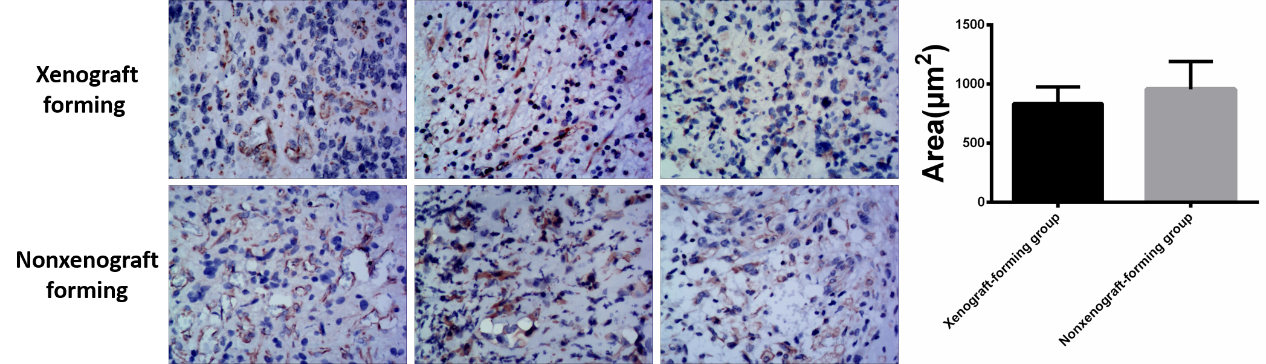
Supplementary material 1.** Immunohistochemical staining of VEGF in glioma surgical specimens. VEGF was positively expressed in tumor vascular hotspots in both groups, but there was no significant difference in the area of the VEGF positive region between the two groups.

|  | **Xenograft forming group** | | **Nonxenograft forming group** | | ***P* Value** | |
| --- | --- | --- | --- | --- | --- | --- |
| Area of the VEGF positive region | | | 835.68 ± 141.02μm^2^ | | 957.31 ± 233.95μm^2^ | 0.158 |

Data are represented as the means ± SD
